# Nosocomial Pathogens: An In-Depth Analysis of the Vectorial Potential of Cockroaches

**DOI:** 10.3390/tropicalmed4010014

**Published:** 2019-01-17

**Authors:** Eric S. Donkor

**Affiliations:** Department of Medical Microbiology, University of Ghana, Accra, Ghana; esampane-donkor@ug.edu.gh; Tel.: +233-55-352-7140

**Keywords:** *Staphylococcus aureus*, nosocomial infections, antibiotic resistance, cockroach

## Abstract

Nosocomial or healthcare-associated infections are regarded as the most frequent adverse event that threatens patients’ safety and has serious economic and social consequences. Cockroach infestation is common in many hospitals, especially in the developing world. Common nosocomial pathogens isolated from cockroaches include *Staphylococcus aureus*, *Pseudomonas aeruginosa*, *Escherichia coli*, and *Klebsiella pneumoniae*. Cockroaches also harbor epidemiologically significant antibiotic-resistant organisms, such as carbapenem-resistant *Enterobacteriaceae*, which complicate nosocomial infections. Therefore, cockroaches constitute an important vector for nosocomial pathogens, and there should be zero tolerance for their presence in healthcare facilities. This paper aims to elucidate the possible role of cockroaches in nosocomial infections by reviewing the relevant research publications.

## 1. Introduction

Cockroaches are adapted to live in various environmental conditions and, consequently, they are found in every part of the world. There exist about 4000 species of cockroach [1], but only 30 are associated with human habitations, including the species *Periplaneta americana*, *Blatella germanica*, and Blatta orientalis [2]. Cockroaches are one of the most significant and objectionable pests. They are common in many human habitations, particularly in places where food is stored or handled. Apart from that, cockroaches are also frequently detected in hospital environments, such as wards and laboratory rooms. In a study of hospital rooms in Poland, *B. germanica* and *B. orientalis* were found in 70% and 40% of hospitals, respectively [3]. Another study at a hospital in Switzerland enumerated about 30 cockroaches (*Ectobius vittiventris*) in a single day, which were found hiding in oxygen masks in the intensive care unit [4]. Cockroaches feed readily on faeces, sputum, skin scrapings, and other human detritus, as well as on a variety of foodstuffs. The filthy behavior of cockroaches coupled with their nocturnal lifestyle make them potential vectors for a wide range of pathogenic microorganisms. The role of cockroaches in human infections had been inconclusive and an issue of debate for several years. Recently, there has been a plethora of research information that contributes significantly to our understanding of this subject. Unfortunately, there are hardly any review papers synthesizing the research data on a single platform to provide a global understanding of the role cockroaches may play in human infections. In this review article, the author aims to describe the significance and possible role of cockroaches in nosocomial infections. 

## 2. Brief Overview of Nosocomial Infections

Nosocomial or healthcare-associated infections are regarded as the most frequent, adverse event that threatens patients’ safety [5,6,7]. For every 100 hospitalized patients at any given time, 7 in developed countries and 10 in developing countries will acquire at least one healthcare-associated infection [8]. The brunt of nosocomial infections appears to be borne by neonates, and the infections are responsible for 4–56% of all deaths caused in this age group [8,9,10]. Based on the anatomical site of infection, the main types of nosocomial infections are urinary tract infections, pneumonia, surgical site infection, and blood stream infections. While urinary tract infections are the most common nosocomial infection in developed countries, surgical site infections are the most common in developing countries, affecting up to one-third of operated patients, and this is up to nine times higher than in developed countries [8,9,10]. A wide range of microbial pathogens including bacteria, parasites, and viruses are implicated in nosocomial infections, though the majority of the infections are associated with bacteria [11,12,13,14]. Nosocomial pathogens tend to differ depending on patient populations, medical facilities, and even differences in the environment in which patient care is administered. Epidemiological evidence indicates that principal nosocomial pathogens include *Staphylococcus aureus*, *Pseudomonas aeruginosa*, *Escherichia coli*, *Acinetobacter baumanii*, and coagulase-negative staphylococcus [8,10,14]. There is also evidence of emerging nosocomial pathogens such as *Cryptosporidium parvum*, *Helicobacter pylori*, and hepatitis C virus [15]. The success of nosocomial pathogens is partly related to their persistence on surfaces in the hospital environment. Relevant nosocomial pathogens such as *S. aureus* and *E. coli* could persist on dry inanimate surfaces for several months [16]. The longer a nosocomial pathogen persists on a surface, the longer it may be a source of transmission in the hospital. Generally, a high inoculum of the nosocomial pathogen, high relative humidity (>70%), and low temperature (around 4 °C) will provide the best chance for long persistence [16,17].

Antibiotic resistance has emerged as a major problem in nosocomial infections and has very serious negative implications for the treatment of such infections, especially in the developing world [18,19,20]. Highly antibiotic-resistant strains frequently encountered in healthcare infections include methicillin-resistant *S. aureus* (MRSA), vancomycin-resistant Enterococci (VRE), carbapenem-resistant *Enterobacteriaceae* (CRE), and extended-spectrum beta-lactamase producing *E. coli* [21]. These bacterial strains account for the majority of outbreaks and mortality cases of healthcare infections. The most studied of these antibiotic-resistant strains appears to be MRSA, which is endemic in many hospitals worldwide, particularly in Europe, the United States, and Asia. In the United States, the annual incidence of invasive MRSA infections in hospitals is estimated to be 94,360, resulting in 18,650 deaths [22,23]. Data from 31 European countries showed that between 2007 and 2008, there were 27,711 episodes of MRSA blood stream infections with 5503 deaths [24]. Though MRSA is considered a nosocomial pathogen traditionally, it has emerged in the community in the last two decades and is responsible for several types of community-acquired infections [25,26,27,28]. Characteristically, healthcare-associated MRSA (HA-MRSA) and community-associated MRSA (CA-MRSA) are considered to be different from each other in terms of their epidemiology, genetic traits, and types of infections [29,30]. Only a few of the known HA-MRSA clones are responsible for the majority of infection, and different clones dominate in different geographical regions. For example, the ST239-SCC*mec*III clone predominates in South American, Asia, and Africa [31,32]. The predominant clone in the United States is CC5-SCC*mec*II (USA100) [33,34], while in Europe it is CC22-SCC*mec*IV (EMRSA-15) [35,36,37,38,39,40]. It is important to note that replacement of these clones keep occurring in several geographical regions [41,42]. Studies on the evolution of the major HA-MRSA clones indicate strong evidence for a wide range of antibiotic-resistant mutations and mobile genetic elements that are associated with the emergence of these clones in hospital epidemics [43,44]. 

Healthcare infections have serious economic and social consequences. These infections take a heavy toll on patients and their families by causing prolonged hospital stays, potential disabilities, and mortality. According to the Centre for Disease Control in the United States, the cost of events related to healthcare-associated infections was, on average, $2100 and varied from $680 for urinary tract infections to $5683 for respiratory tract infections [45]. In the United Kingdom, healthcare-associated infections cost about £1 billion a year; £56 million is estimated to incur after patients are discharged from hospital [46]. In the developing world, information on costs related to healthcare-associated infections is limited and available only in a few countries. A study from Thailand reported that the extra costs associated with surgical site infections was U.S. $1091 [47], while another study in Turkey showed that extra cost for a patient with a nosocomial infection was U.S. $2026 [48]. The huge public health impact of nosocomial infections coupled with the associated financial costs underscores the need for effective prevention of these infections. This requires good hygienic practices including hand-washing of healthcare workers and disinfection of surfaces in the hospitals, microbiological surveillance of nosocomial pathogens using standard laboratory protocols, applying barrier precautions to high-risk patients, judicious use of antibiotics in the hospital, limiting the duration of use of intravascular catheters [17,21,45].

## 3. Evidence for the Role of Cockroaches in Human Infections

Compelling evidence incriminating cockroaches as vectors of human infections comes from a study by Tarshis [49] in the 1950s, which reported a correlation between lack of cockroach control and incidence of infectious hepatitis in Los Angeles, Southern California. Between 1956 and 1959, the Carmelitos Housing Project had 20 to 39% of hepatitis A cases in Los Angeles. However, in 1960, the incidence of hepatitis A at the housing project dropped to 6.6%, then further to 3.6% in 1961, and to 0.0% in 1962. It was observed that the drop in the incidence of hepatitis A occurred concurrently with about a 70% drop in the infestations of *B. germanica*, *B. orientalis*, and *Supella longipalpa* cockroaches at the Carmelitos Housing Project, which was the consequence of a concentrated pest control program. Around the same time, the incidence of the infection had been rising everywhere else in Los Angeles County that was not receiving the pest control service. The association of cockroaches with the hepatitis A epidemic in Los Angeles is interesting and demonstrates the huge extent of these insects as vectors of infectious agents implicated in human infections. The hepatitis A virus is present in the faeces of infected persons and is most often transmitted through consumption of contaminated water or food. The association of cockroaches with faeces and food makes their transmission of hepatitis A virus highly plausible. In the hepatitis A epidemic in Los Angeles, cockroaches traversing between the sewage system and the houses were pinpointed as the source of the epidemic.

Major experimental evidence supporting the role of cockroaches as vectors of microbial pathogens was found in a study by Kopanic et al. [50] in 1993. In that study, experiments were conducted to find out if individual cockroaches could acquire a naladixic acid-resistant strain of *Salmonella Typhimurium* from a contaminated food source and then transfer this microbe to uninfected cockroaches, food (eggs), and water. Cockroaches, food, and water were sampled after 24, 48, 72, and 96 hours and tested for *S. Typhimurium*. All three types of samples were positive for *S. Typhimurium* at each 24 h sampling period. The highest frequency of *S. Typhimurium* cross-contamination between infected and uninfected cockroaches occurred within 24 h and declined thereafter. Water sites were heavily contaminated throughout the 96 h test period. Eggs that had been exposed for 24 h to infected cockroaches contained a minimum of 75 *S. Typhimurium* cells per egg. These findings clearly show the vectorial potential of cockroaches in the contamination of food and water and their role as important agents of human infection. 

In 2003, Imamura et al. [51], using *H. pylori*, provided an additional piece of experimental evidence implicating cockroaches as vectors of microbial pathogens. After a three-day fast, cockroaches were divided into two groups; one group was transferred onto agar plates of freshly grown *H. pylori* (challenge group), while the other was placed on sterile agar plates without *H. pylori* (control group). After 24 h, the cockroaches were transferred to disinfected containers and provided with sterile food and water. The external surfaces and excreta of the cockroaches were sampled for microbiological examination by culture, rapid urease test, and polymerase chain reaction. The investigators detected *H. pylori* in both excreta and external surfaces of the challenged group, though at higher levels in the excreta. In the excreta of the challenged group, *H. pylori* was culturable for a 24 h post-challenge, the urease test was positive for up to day 3, and the PCR analysis was positive for up to day 7. All of the controlled cockroach samples were negative for *H. pylori*.

## 4. Cockroaches and Nosocomial Pathogens

Cockroaches pose a concern in the hospital environment because they may serve as reservoirs and vectors for nosocomial pathogens. Microbial carriage of cockroaches in hospitals has been reported by several studies (Table 1) [52,53,54,55,56,57,58,59,60,61,62]. Generally, these studies seem to have focused on bacteria and, to some extent, parasites and fungi, while very little has been done on viruses. A study reported 19.7% carriage of rotavirus among cockroaches collected at a tertiary hospital in Ghana [61]. Rotavirus is the most common cause of severe and fatal diarrhea in young children worldwide and accounts for about half of the diarrheal disease hospitalizations of children under five years of age in developing countries [61,63,64,65,66,67]. Moreover, nosocomial rotavirus infection seems to be responsible for about 25% of all rotavirus-related hospitalizations, especially in immune-compromised children [68,69]. Thus, the occurrence of cockroaches in hospitals and homes could have serious health implications for children. As shown in Table 1, parasites carried by cockroaches that were sampled in hospitals are predominantly those implicated in gastroenteritis. Some of these gastrointestinal parasites are also implicated in nosocomial infections, suggesting that cockroaches could play a role in nosocomial parasitosis. For instance, *Entamoeba histolytica* is frequently implicated in parasitic nosocomial epidemics involving diarrhea [70,71,72], while *Entamoeba coli* and *Endolimax nana* are detected most frequently in nosocomial parasitosis involving transplantation [72]. Nosocomial invasive fungal infections are usually caused by *Candida* and *Aspergillus* [73,74]. Both fungi were reported to be carried by cockroaches sampled in the hospital environment [56,58]. This indicates that cockroaches may also have some relevance in nosocomial mycoses. A wide range of bacteria have been isolated from cockroaches in hospitals (Table 1). In one study, as many as 174 bacteria were isolated [60]. This highlights the close association of cockroaches and bacteria, which is not surprising given the broad habitat range of cockroaches and the ubiquitous nature of bacteria. As shown in Table 1, the bacterial florae isolated from cockroaches in different countries in the developed and developing world, appear to be similar. This suggests that the type of bacterial flora carried by cockroaches may be independent of geographical location or socio-economic factors. Several of these bacterial agents including *S. aureus*, *E. coli*, *P.s aeruginosa*, and *Klebsiella pneumoniae* are recognized nosocomial pathogens. For example, *K. pneumoniae* and *S. aureus* are leading causes of nosocomial pneumonia, while *E. coli* is the commonest cause of nosocomial urinary tract infection [11,14,21].

## 5. Cockroaches and Antibiotic Resistance

In many instances, cockroaches have been reported to carry highly antibiotic-resistant bacteria, therefore, it is important to highlight these studies. A study in Ethiopia showed that 64.1% of bacteria isolated from cockroaches were multidrug-resistant (resistance to three or more classes of antibiotics); *Salmonella* spp. were the most prevalent multidrug-resistant isolates (100%) followed by *Enterobacter* (90.5%) and *Shigella* spp. (76.9%) [57]. Interestingly, in that study the bacteria isolated from cockroaches in the hospital environment had a higher prevalence of multidrug resistance (67%) compared to those from the community (61.3%) [57]. The risk of cockroaches as reservoirs of antibiotic-resistant bacteria in the hospital environment has been elucidated by another study carried out at the neonatal intensive care unit of a hospital in Ethiopia [62]. In that study, the key nosocomial pathogens implicated in neonatal sepsis at the study hospital, including *K. pneumoniae* and *Klebsiella oxytoca*, were isolated from the cockroaches, and these organisms were predominantly multidrug-resistant [72]. A study in a hospital in Iraq showed that about 42.8% of cockroach bacterial isolates were found to express the extended-spectrum beta-lactamases (ESβLs) trait, while 19% expressed the metallo-β-lactamases (MβLs) trait [75]; both ESβLs and MβLs are enzymes that confer resistance to a broad range of β-lactam drugs. Studies in Ghana and Algeria showed that household and hospital cockroaches could serve as reservoirs of the *CTX-M-15*, *OXA-48*, and *NDM-1* genes that share beta-lactam resistance determinants with humans [76,77]. The cockroach brain has antimicrobial properties, and this is thought to be an important factor that accounts for the carriage of antibiotic-resistant organisms among cockroaches [78]. In hospitals, cockroaches that carry antibiotic-resistant bacteria could easily disseminate these organisms on hospital equipment and, therefore, facilitate their transmission to patients. This implies that cockroaches could play a significant role in outbreaks of nosocomial pathogens in hospitals, though little attention has been given to this. In an outbreak of *K. pneumoniae* in a neonatal unit infested with cockroaches in South Africa, the organisms isolated from cockroaches and those colonizing infants or causing clinical disease were indistinguishable by pulsed-field gel electrophoresis [79]. This suggests the possibility of some transmission events between cockroaches and humans, though the investigators needed whole-genome sequencing analysis to confirm this. Antibiotic resistance is considered one of the greatest threats to public health in recent times, and, therefore, cockroach infestation should be of a serious concern, given the possible role of cockroaches as reservoirs of antibiotic-resistant bacteria. 

## 6. Conclusions and Further Research

Cockroaches could harbor and disseminate many microbial pathogens including bacteria, fungi, viruses, and parasites. Since many of these microorganisms are implicated in healthcare-associated infections, the presence of cockroaches in the hospital environment constitutes an important reservoir of vectors of nosocomial pathogens. In addition, cockroaches tend to serve as reservoirs of antibiotic-resistant organisms, which complicates healthcare-associated infections.

Despite cockroaches being reservoirs and vectors of nosocomial pathogens, their involvement in the actual transmission of these pathogens to patients in hospitals has not been proven. The era of whole-genome sequencing provides us with the opportunity to address this research gap. In hospitals, whole-genome sequencing of nosocomial pathogens isolated from cockroaches, patients, healthcare workers, hospital equipment, and other fomites could provide insights into the transmission of nosocomial pathogens involving cockroaches. There is also the need for further studies to describe cockroach carriage of epidemiologically significant antibiotic-resistant organisms, such as MRSA and VRE, which have not been previously reported. In addition, studies are required to elucidate the possible mechanisms of host–pathogen relationships that may exist between cockroaches and microbial pathogens.

Considering the microbial risk to human health associated with cockroaches, there should be zero tolerance for their presence in healthcare facilities. Control of cockroaches in healthcare facilities could involve proper sanitation of equipment and facilities to remove dirt and food debris, as well as the application of insecticides to hiding places of cockroaches [80,81,82]. There is also the need to discard cardboard and to keep food in tightly fitted containers. It is believed that the effective control of cockroaches will substantially minimize the spread of infectious diseases in our environment [83].

## Figures and Tables

**Table 1:** Microbial Carriage of Cockroaches in Healthcare Institutions.

Study	Country	N	Organisms Isolated
Fotedar et al. [52]	India	96	*Pseudomonas aeruginosa*, *Staphylococcus aureus*, *Streptococcus faecalis*, *Micrococcus* spp.
Tachbele et al. [53]	Ethiopia	600	*Salmonella* spp., *Shigella flexneri*, *Escherichia coli O157*, *Staphylococcus aureus* and *Bacillus cereus*.
Oothuman et al. [54]	Malaysia	104	*Shigella boydii*, *Shigella dysenteriae*, *Salmonella Typhimurium*, *Klebseilla oxytoca*, *Klebsiella ozaena*, *Serratia marcescens*.
Guyader et al. [55]	France	532	*Citrobacter freundii*, *Enterobacter cloacae*, *Klebsiella oxytoca*, *Klebsiella pneumoniae*, *Enterobacter agglomerans*, *Escherichia adecarboxylata*, *Serratia marcescens*, *Serratia liquefaciens*, *Acinetobacter* spp., *Pseudomonas fluorescens*, *Pseudomonas putida*, *Pseudomonas aeruginosa*, *Escherichia coli*, *Staphylococcus aureus*.
Fotedar et al. [56]	India	279	Bacteria: *Klebsiella* spp., *Escherichia coli*, *Enterobacter* spp., *Pseudomonas aeruginosa*, *Proteus* spp., *Staphylococcus aureus*, *Streptococcus epidermidis*, *Streptococcus faecalis*, *Streptococcus viridans*, *Micrococcus*, *Bacillus* spp. Parasites: *Endolimax nana*, *Entamoeba coli*, *Entamoeba histolytica*. Fungi: *Candida* spp., *Rhizopus* spp. *Mucor* spp., *Alternaria* spp. *Aspergillus niger*, *Aspergillus flavus*, *Aspergillus* spp.
Moges et al. [57]	Ethiopia	60	*Klebsiella pneumoniae*, *Escherichia coli*, *Citrobacter* spp., *Salmonella* spp., *Enterobacter* spp. *Shigella* spp., *Providencia* spp., *Serratia* spp., *Proteus* spp., *Staphylococcus aureus*, *Escherichia coli.*
Salehzadeh et al. [58]	Iran	133	Bacteria: *Enterobacter* spp., *Klebsiella* spp., *Enterococcus* spp., *Staphylococcus* spp., *Escherichia coli*, *Streptococcus* spp., *Pseudomonas* spp. *Shigella* spp, *Haemophilus* and group A beta-hemolytic *Streptococcus* spp. Fungi: *Candida* spp., *Mucor* spp., *Rhizopus* spp., *Penicillium* spp., *Aspergillus fumigans*. *Aspergillus niger*.
Naher et al. [59]	Bangladesh		*Escherichia coli*, *Pseudomonas aeruginosa*, *Salmonella* spp., *Shigella* spp., *Klebsiella* spp., *Proteus* spp., *Staphylococcus aureus*.
Mensaria et al. [60]	Algeria		*Citrobacter freundii*, *Enterobacter cloacae*, *Serratia marcescens*, *Klebsiella pneumoniae*, *Pantoea* spp., *Enterobacter aerogenes*, *Enterobacter* spp.
Tetteh-Quarcoo et al. [61]	Ghana	61	Bacteria: *Klebsiella pneumoniae*, *Escherichia coli*, *Proteus vulgaris*, *Citrobacter ferundii*, *Enterobacter cloacae*, *Enterococcus faecalis*, *Pseudomonas aeruginosa*, *Klebsiella oxytoca*. Parasites: *Ancylostoma duodenale*, *Hymenolepis nana*, *Taenia* spp. Viruses: Rotavirus.
Tilahun et al. [62]	Ethiopia	400	*Klebsiella oxytoca*, *Klebsiella pneumoniae*, *Klebsiella ozaenae*, *Citrobacter* spp., *Citrobacter diversus*, *Enterobacter cloacae*, *Pseudomonas aeruginosa*, *Enterobacter aeruginosa*, *Providencia rettgeri*, *Salmonella* spp., *Streptococcus* spp. *Staphylococcus aureus*, *Escherichia coli*, *Acinetobacter* spp. and *Shigella flexneri*

N, number of cockroach samples.
